# Impact of concurrent lobular carcinoma in situ on recurrence outcomes in patients with classic and pleomorphic invasive lobular carcinoma of the breast

**DOI:** 10.1007/s10549-025-07864-7

**Published:** 2025-11-27

**Authors:** Mandeep Kaur, Astrid Quirarte, Anna Vertido, Taron Torosian, Thomas M. Li, Jason A. Mouabbi, Rita A. Mukhtar

**Affiliations:** 1 School of Medicine, University of California, San Francisco, CA USA; 2 Division of Surgical Oncology, Department of Surgery, University of California, San Francisco, CA USA; 3 Department of Surgery, University of California, San Francisco, CA USA; 4 Department of Breast Medical Oncology, The University of Texas MD Anderson Cancer Center, Houston, TX USA

**Keywords:** Invasive lobular carcinoma, Lobular carcinoma in situ, Lobular carcinoma

## Abstract

**Purpose:**

We investigated outcomes of invasive lobular carcinoma (ILC) with or without concurrent lobular carcinoma in situ (LCIS) in patients with classic or pleomorphic ILC.

**Methods:**

We retrospectively analyzed a single-institution database of patients with stage I-III ILC. We compared tumor features, treatment, and recurrence free survival (RFS) in patients with ILC-alone versus ILC + LCIS stratified by ILC tumor subtype. Multivariable Cox proportional hazards models were used for multivariate analysis.

**Results:**

Of the 786 cases of ILC, 542 were classic and 92 were pleomorphic, with 70.6% overall having concurrent LCIS. Overall, ILC + LCIS cases were less often T3 (p = 0.037) and had lower rates of N2/N3 disease (p = 0.026) than ILC-alone. Concomitant LCIS was also associated with greater progesterone receptor (PR) positivity (p = 0.016), and was more commonly grade 2 and less often grade 1 compared to ILC-alone (p = 0.008). Treatment differed, with ILC + LCIS cases receiving less chemotherapy (p = 0.016) and more mastectomy (p = 0.015). Among patients with classic ILC, the presence of concomitant LCIS was not associated with different RFS. However, among those with pleomorphic ILC, ILC + LCIS was associated with significantly improved RFS compared to ILC-alone (HR 0.31, 95% confidence interval 0.10–0.96, p = 0.043).

**Conclusion:**

While the presence of LCIS was not associated with RFS in classic ILC in this dataset, it is a favorable prognostic factor in pleomorphic ILC, suggesting a potentially differential role in ILC subtypes.

## Introduction

Invasive lobular carcinoma (ILC) is the second most common histologic subtype of breast cancer, accounting for 10–15% of all cases [1]. Compared to the more common invasive ductal carcinoma (IDC), ILC has distinct genomic and clinical features that impact its diagnosis and outcomes [2]. Because it lacks the adhesion protein E-cadherin, ILC grows in a diffuse pattern in so-called single files lines [3, 4]. This unique growth pattern can impact the sensitivity of imaging tools, leading to delays in diagnosis and presentation at more advanced stages [5, 6]. Additionally, surgical treatment of ILC is fraught with higher rates of positive margins and need for more extensive surgery [7–9], while optimal systemic therapy selection can be challenging because of high rates of discordance between clinical risk and molecular risk in this tumor type [10, 11].

Accordingly, understanding the drivers of ILC development could allow for identification of patients at risk for ILC and tailoring screening [12]. Additionally, appreciation for the heterogeneity within ILC and identification of ILC-specific risk markers could select patients for more appropriate therapy. For example, ILC can be further classified into subtypes, including classic and pleomorphic, with pleomorphic cases having higher grade and worse outcomes [13–15]. Recently, the presence of concomitant lobular carcinoma in situ (LCIS) has been identified as a potential prognostic factor that could be included in risk stratification for patients with ILC[16–18].

The biological role and clinical significance of LCIS is incompletely understood [19–22]. When found in isolation (without a concomitant invasive component), LCIS is typically thought of as a risk factor for the development of future breast cancer in either breast [23]. As such, patients with LCIS may be offered risk reduction and high-risk screening. However, LCIS is commonly identified concomitantly with invasive tumors, with studies showing that up to half of ILC tumors contain a component of LCIS [24]. Studies evaluating genomic features of concomitant LCIS with ILC suggest clonal similarities in many cases, suggesting that LCIS may be a non-obligate precursor to ILC [25–27]. Consequently, the presence or absence of LCIS in an invasive lobular tumor may reflect differences in tumor development and biology. Two prior studies found that the presence of concomitant LCIS in those with ILC was associated with improved prognosis compared to those with ILC alone, but whether this is true among ILC subtypes in unknown[28, 29]. In this investigation of a single-institution cohort, we evaluated whether the presence of concomitant LCIS in ILC is associated with differential outcomes in patients with classic or pleomorphic ILC.

## Methods

With Institutional Review Board approval, we analyzed a prospectively maintained institutional database containing surgical outcomes for all consecutive patients treated for ILC at the University of California, San Francisco between January 1996 and September 2019.

Clinicopathologic features including subtype of ILC, presence or absence of LCIS, tumor stage, tumor receptor subtype, presence of lymphovascular invasion (LVI), tumor grade, multifocality, and presence of contralateral malignancy were collected. Subtypes of ILC assessed include classic, pleomorphic, mixed ILC/IDC, and other. Presence of LCIS was determined recorded from surgical pathology reports and grouped as present or absent in our analysis. In this analysis tumors without LCIS are termed ILC-alone, and tumors with concurrent LCIS are termed ILC + LCIS. Nodal status was grouped as N0/N1 versus N2/N3. Tumor receptor subtype was classified by estrogen receptor (ER), progesterone receptor (PR), and human epidermal growth factor 2 (HER2) status, and grouped as hormone receptor (HR) positive/HER2 negative, HR negative/HER2 negative, and HER2 positive.

Patients with concurrent IDC were included, but those with de novo stage IV disease, missing LCIS data, and missing surgical treatment data were excluded from the analysis. Data on prior malignancies were not collected.

### Statistical analysis

Data were analyzed in Stata 17.0 using chi-squared tests, t-tests, and logistic regression models to evaluate associations between pure ILC, concurrent ILC and LCIS, subtype of ILC, characteristics, and treatment in patients with stage I-III ILC. Recurrence events included local and/or distant recurrence, and patients were censored at the time of event or last follow up if no recurrence event occurred. We compared recurrence free survival (RFS) between groups using the log rank test and used Cox proportional hazards models for multivariate analyses. The multivariate model included presence of concurrent LCIS, tumor size, number of positive nodes, and tumor receptor subtype. Data are reported with 95% confidence intervals (CI) and two-tailed p-values < 0.05 considered significant.

## Results

### Study cohort

We identified 786 cases of stage I-III ILC in our institutional cohort. Of these, 542 were classic (69%), 92 were pleomorphic (12%), 57 were mixed ILC/IDC (7.3%), and 95 were other (12%) subtypes of ILC. Overall, 71% of the study cohort had concurrent LCIS. Concurrent LCIS was significantly more common in classic and pleomorphic ILC cases than mixed ILC/IDC (concurrent LCIS present in 70% of classic ILC, 79% of pleomorphic ILC, and 51% mixed ILC/IDC, p = 0.001). Compared to ILC-alone, tumors with concurrent LCIS were significantly smaller, in both the classic and pleomorphic subtypes, and had less advanced nodal involvement overall (Tables 1, 2, 3). There were no differences in receptor subtype group overall, but tumors with concurrent ILC + LCIS were significantly more often PR positive than ILC-alone tumors (84% versus 76%, p = 0.016). Compared to non-pleomorphic cases, pleomorphic subtypes were less often HR + and more often triple negative or HER2 + (HR + 68% versus 95%, triple negative 14% versus 1.5%, HER2 + 18% versus 3.8%, p < 0.01). Tumors with concurrent ILC + LCIS were more commonly grade 2 (70% versus 59%, p = 0.008), and less often grade 1 (24% versus 35%, p = 0.008) than ILC-alone.

**Table 1:** ILC characteristics by overall subtype of ILC

Characteristic	Study cohort (N = 786)	ILC Alone (n = 231)	ILC + LCIS (n = 555)	p-value
Age (mean ± SD)	59.7 ± 12.1	60.7 ± 12.6	59.3 ± 11.9	0.1305
Tumor Size, cm (mean ± SD)^1^	3.2 ± 2.9	3.4 ± 3.3	3.1 ± 2.7	< 0.001
T Stage^1^				0.037
1	48%	47%	48%	
2	32%	27%	34%	
3	21%	26%	19%	
N Stage^2^				0.026
N0/N1	89%	86%	91%	
N2/N3	11%	14%	9.1%	
Receptor Subtype^3^				0.16
HR +/HER2-	92%	92%	92%	
HR-/HER2-	2.9%	2.7%	3.0%	
HER2 +	5.3%	5.8%	5.1%	
PR Positivity^4^	81%	76%	83%	0.016
Tumor Grade^5^				0.008
1	27%	35%	24%	
2	67%	59%	70%	
3	5.5%	5.8%	5.3%	

Data reported from complete case analyses. *SD* Standard deviation, *HR* Hormone receptor, *HER2* Human epidermal growth factor receptor 2, + Receptor status positive, − Receptor status negative. ^1^ n = 779, ^2^ n = 780, ^3^ n = 732, ^4^ n = 775, ^5^ n = 770.

**Table 2:** ILC characteristics by classic ILC

Characteristic	Classic ILC			p-value
	Total (n = 542)	ILC Alone (n = 164)	ILC + LCIS (n = 378)	
Age (mean ± SD)	59.4 ± 11.4	59.7 ± 11.6	59.3 ± 11.3	0.656
Tumor Size, cm (mean ± SD)^1^	3.1 ± 3.0	3.3 ± 3.3	3.0 ± 2.8	0.032
T Stage^1^				0.131
1	50%	48%	51%	
2	31%	28%	32%	
3	19%	24%	17%	
N Stage^2^				0.091
N0/N1	89%	85%	92%	
N2/N3	11%	15%	8.4%	
Receptor Subtype^3^				0.513
HR +/HER2−	95%	96%	94%	
HR−/HER2−	1.6%	1.3%	1.7%	
HER2 +	3.7%	3.1%	4.0%	
PR Positivity^4^	82%	78%	84%	0.116
Tumor Grade^5^				0.044
1	34%	41%	31%	
2	65%	58%	68%	
3	1.1%	1.9%	0.8%	

Data reported from complete case analyses. *SD* Standard deviation, *HR* Hormone receptor, *HER2* Human epidermal growth factor receptor 2, + Receptor status positive, − Receptor status negative. ^1^ n = 537, ^2^ n = 539, ^3^ n = 508, ^4^ n = 535, ^5^ n = 529.

**Table 3:** ILC characteristics by pleomorphic ILC

Characteristic	Pleomorphic ILC			p-value
	Total (n = 91)	ILC Alone (n = 19)	ILC + LCIS (n = 72)	
Age (mean ± SD)	60.5 ± 13.3	63.3 ± 14.8	59.8 ± 12.9	0.45
Tumor Size, cm (mean ± SD)^1^	3.6 ± 3.2	5.1 ± 4.6	3.2 ± 2.7	0.0187
T Stage^1^				0.017
1	40%	37%	40%	
2	36%	16%	42%	
3	24%	47%	18%	
N Stage^2^				0.286
N0/N1	82%	67%	86%	
N2/N3	18%	33%	14%	
Receptor Subtype^3^				0.574
HR +/HER2−	68%	56%	72%	
HR−/HER2−	14%	22%	12%	
HER2 +	18%	22%	16%	
PR Positivity^4^	66%	47%	71%	0.049
Tumor Grade^5^				0.393
1	1.1%	0%	1.4%	
2	69%	58%	72%	
3	30%	42%	27%	

Data reported from complete case analyses. *SD* Standard deviation, *HR* Hormone receptor, *HER2* Human epidermal growth factor receptor 2, + Receptor status positive, − Receptor status negative. ^1^ n = 91, ^2^ n = 90, ^3^ n = 79, ^4^ n = 89, ^5^ n = 90.

Overall, 86 patients had a contralateral malignancy (11%), and this was less common in the pleomorphic subtypes when compared to others (2.3% versus 98%, p = 0.004). Among the classic subtype, ILC + LCIS was associated with higher tumor multifocality (40% versus 21%, p < 0.01). This finding was not true for the pleomorphic subtype (21% versus 37%, p = 0.157).

### Treatment

Patients with concurrent ILC + LCIS received less chemotherapy and neoadjuvant endocrine therapy (Table 3, 4). This was true in both the classic and pleomorphic subtypes (Tables 5 and 6). Compared to non-pleomorphic cases, pleomorphic subtypes were more likely to receive chemotherapy (59% versus 33%, p < 0.01). Those with concurrent ILC + LCIS received more mastectomy (53% versus 48%, p = 0.015), and less breast conserving therapy (47% versus 52%, p = 0.015). While this was true in the classic subtype (54% mastectomy for ILC + LCIS versus 46% ILC alone, and 46% BCT for ILC + LCIS versus 54%, p = 0.01), this was not demonstrated in the pleomorphic subtype (49% mastectomy for ILC + LCIS versus 59% ILC alone, and 51% BCT for ILC + LCIS versus 42%, p = 0.063). Multifocal tumors were also associated with higher mastectomy rates compared others (44% versus 33%, p = 0.001).

**Table 4:** Treatment for ILC overall

Characteristic	ILC Overall	ILC alone	ILC + LCIS	p-value
Neoadjuvant endocrine therapy^1^	13%	23%	8.7%	< 0.001
Chemotherapy^1^	36%	42%	33%	0.016
Mastectomy^2^	51%	48%	53%	0.015
Breast conserving therapy^2^	49%	52%	47%	0.015

Data reported from complete case analyses. *SD* Standard deviation, *HR* Hormone receptor, *HER2* Human epidermal growth factor receptor 2, + Receptor status positive, − Receptor status negative. ^1^ n = 780, ^2^n = 777.

**Table 5:** Treatment by classic ILC subtype

Characteristic	Classic ILC		p-value
	ILC alone	ILC + LCIS	
Neoadjuvant endocrine therapy^1^	22%	8.5%	< 0.001
Chemotherapy^1^	41%	30%	0.015
Mastectomy^2^	46%	54%	0.01
Breast conserving therapy^2^	54%	46%	0.01

Data reported from complete case analyses. *SD* Standard deviation, *HR* Hormone receptor, *HER2* Human epidermal growth factor receptor 2, + Receptor status positive, − Receptor status negative. ^1^ n = 539, ^2^ n = 538.

**Table 6:** Treatment by pleomorphic ILC subtype

Characteristic	Pleomorphic ILC		p-value
	ILC alone	ILC + LCIS	
Neoadjuvant endocrine therapy	68%	11%	< 0.001
Chemotherapy	84%	53%	0.013
Mastectomy	58%	49%	0.063
Breast conserving therapy	42%	51%	0.063

Data reported from complete case analyses. *SD* Standard deviation, *HR* hormone receptor, *HER2* Human epidermal growth factor receptor 2, + Receptor status positive, − receptor status negative. n = 91.

### Recurrence free survival

**Fig. 1:**
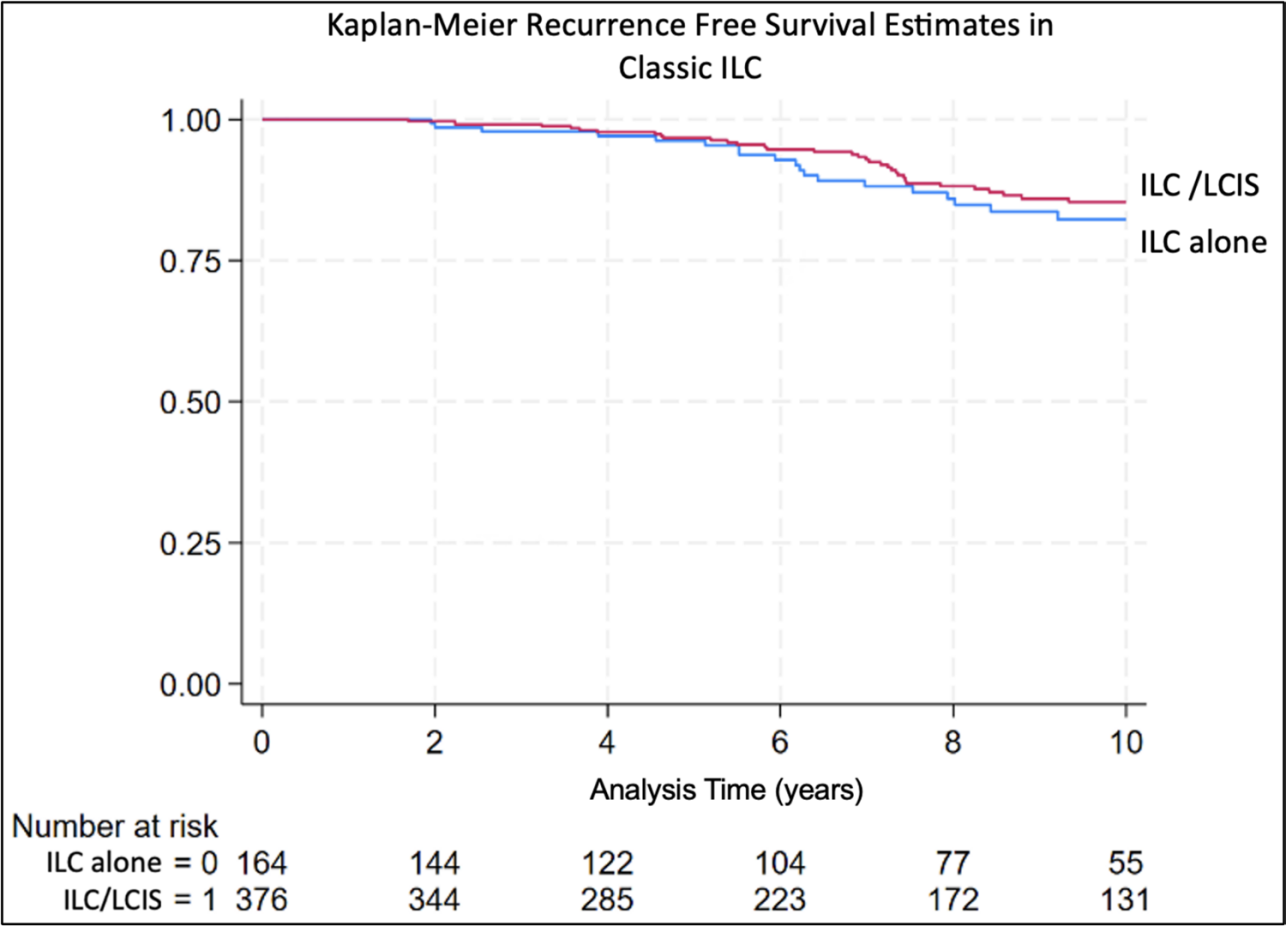
Kaplan–Meier recurrence free survival estimate in classic ILC group

**Fig. 2:**
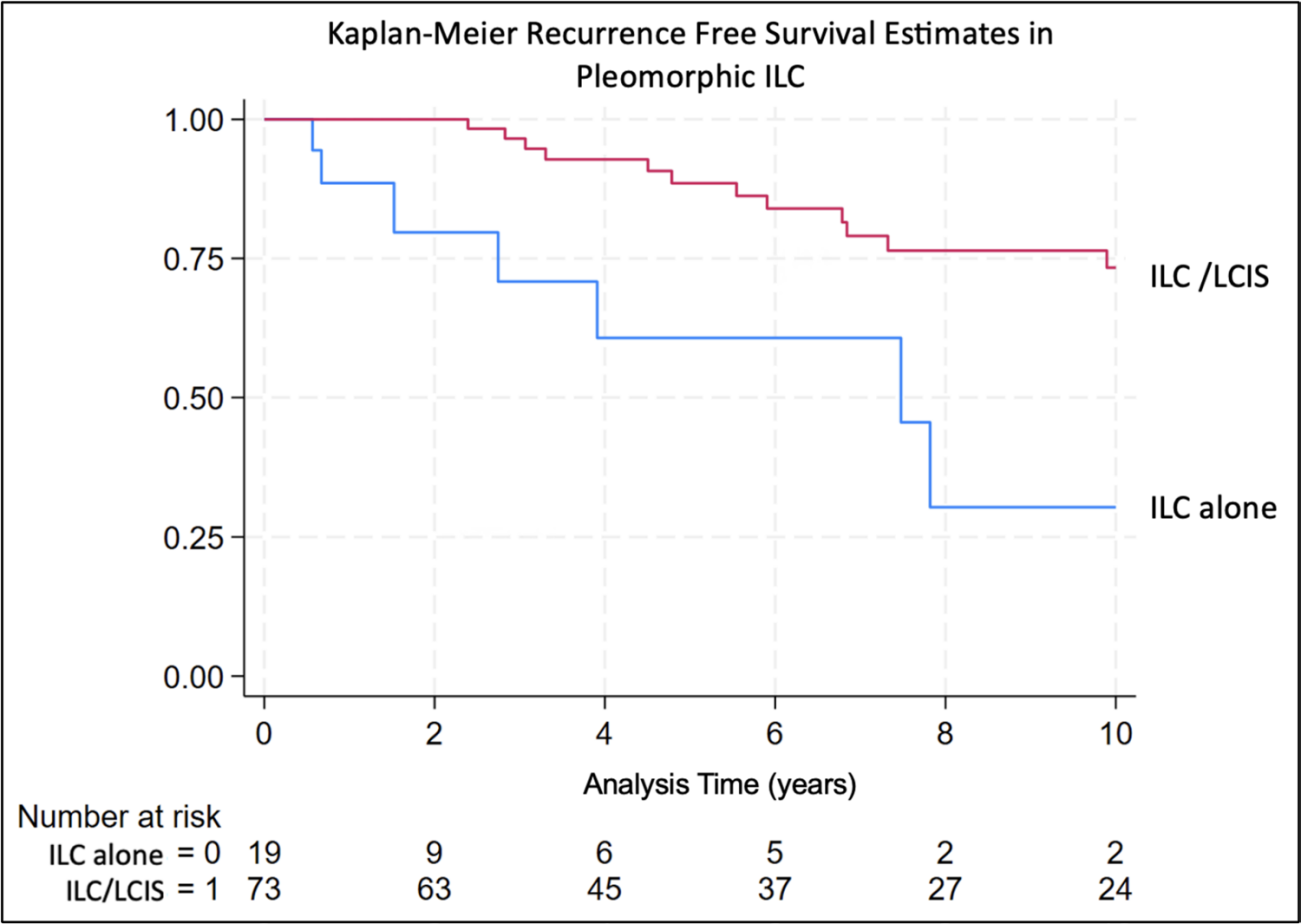
Kaplan–Meier recurrence free survival estimate in pleomorphic ILC group

There were 72 recurrence events in the study population (53 among 540 patients with classic ILC and follow up data, and 19 among 92 patients with pleomorphic ILC and follow up data). On univariate analysis, there was no significant difference in RFS between ILC-alone and concurrent ILC + LCIS in those with classic ILC (Fig. 1). However, among those with pleomorphic ILC, those with concurrent ILC + LCIS had significantly improved RFS compared to ILC-alone (Fig. 2).

In a multivariable model including tumor size, number of positive nodes, receptor subtype, and treatment type, patients with concurrent ILC + LCIS had significantly improved RFS compared to those with ILC-alone among patients with pleomorphic subtype (HR 0.15, 95% confidence interval (CI) 0.04–0.62.04.62, p < 0.009, Table 7).

**Table 7:** Multivariable cox proportional hazards model for RFS among pleomorphic ILC cases, adjusting for tumor size, number of positive nodes, receptor subtype, and treatment type

Variables	Hazard ratio	95% Confidence interval	p-value
Pleomorphic ILC alone (reference)			
Pleomorphic ILC with LCIS	0.15	0.04–0.62	0.009
Tumor size (cm)	1.33	1.05–1.68	0.019
Number of positive nodes	1.04	0.97–1.11	0.298
Subtype			
ER +/PR +/HER2- (reference)			
ER +/PR-/HER2-	4.34	0.38–49.65	0.238
Triple negative	9.29	1.49–58.16	0.017
HER2 +	2.49	0.48–12.82	0.276
Treatment			
Local radiation (reference)			
Lumpectomy	5.99	0.99–36.41	0.052
Mastectomy	0.77	0.14–4.28	0.762
Mastectomy and radiation	0.24	0.02–2.50	0.231
Chemotherapy	1.05	0.20–5.60	0.951

## Discussion

Our findings reveal that concurrent LCIS is common in both classic and pleomorphic ILC subtypes, and is generally associated with more favorable prognostic features, such as fewer T3 tumors, less advanced nodal involvement, and more PR + cases. Consequently, ILC + LCIS is associated with less chemotherapy and less neoadjuvant endocrine therapy use. Despite this, mastectomy rates were higher in the patients with concurrent LCIS, although the drivers behind this are unclear since ILC + LCIS cases were smaller in size. There was no difference in RFS in those with classic ILC, but among those with pleomorphic subtype, the presence of concurrent LCIS was associated with significantly improved RFS.

LCIS and ILC share genomic similarities. For example, CDH1 mutations are present in 56% of LCIS and 66% of ILC, and PIK3CA occur in 41% of LCIS and 52% of ILC lesions [30]. Other commonly shared recurrent somatic mutations occur in ERBB2, ERBB3, FOXA1, and TP53 [31]. Loss of heterozygosity is also found at chromosome 11q13 in both LCIS and ILC, particularly when LCIS is associated with ILC [32]. This suggests that in some cases, LCIS is thought to be a precursor to ILC [25, 33, 34].

Prior studies have shown that concurrent LCIS is a good prognostic indicator for patients with ILC in general [28, 29]. Our findings were consistent with this among the pleomorphic subset of patients, but not among those with classic ILC. For those with pleomorphic ILC, the risk of recurrence is higher than those with classic ILC [35]. Pleomorphic ILC is generally defined by the presence of high nuclear pleomorphism, and consequently these tumors tend to be of higher grade [36, 37]. Additionally, pleomorphic ILC is more likely to have ER negativity or HER2 amplification [15, 36]. Because of the high risk features associated with this histologic diagnosis, patients with pleomorphic ILC are more often treated with cytotoxic chemotherapy than those with classic ILC, which was consistent with our results [38, 39]. Our findings may help risk stratify these patients, since the presence of concomitant LCIS appears to identify a subset of pleomorphic ILC patients with significantly improved outcome, even after accounting for factors such as contralateral malignancy and multifocality. Other factors that appear to impact outcome in patients with pleomorphic ILC is mitotic score, which contributes to overall grade [39, 40]. Further evaluation of these cases is needed.

These findings have important clinical implications, as improved understanding of the heterogeneity within lobular tumors may help personalize treatment. However, these results should be viewed within the context of the limitations of this study. Firstly, the retrospective design of this study may introduce selection bias, and the cohort resulting from a single institution may limit generalizability and introduce both selection and treatment biases. Secondly, while the cohort was large overall, the number of patients with pleomorphic ILC and recurrence events was relatively small, which may limit the power of subgroup analyses. Finally, while we were able to capture features such as contralateral malignancy and multifocality for a subset of our population, data on imaging findings and other specifics were not available uniformly and could have impacted treatment decision-making patterns.

Overall, our findings contribute to the broader understanding of lobular neoplasia. The relationships between LCIS and ILC remains incompletely understood. Additionally, the clinical implications of classic versus non-classic ILC, and whether those subtypes should drive treatment, is an area of active debate [35, 39, 41, 42]. For those patients with ILC and the highest risk of recurrence, such as pleomorphic cases, consideration of other prognostic factors, such as the presence of LCIS, may help tailor treatment strategy.

Model included pleomorphic ILC cases, Tumors size, Number of positive nodes, and Receptor subtype. *ER* Estrogen receptor, *PR* Progesterone receptor, *HER2* Human epidermal growth factor receptor 2, + Receptor status positive, − Receptor status negative. Data available in n = 76.

## Data Availability

The datasets generated during and/or analyzed during the current study are not publicly available but are available from the corresponding author upon reasonable request.
